# Clinical, Biological, Radiological Pathological and Immediate Post-Operative Remission of Sparsely and Densely Granulated Corticotroph Pituitary Tumors: A Retrospective Study of a Cohort of 277 Patients With Cushing’s Disease

**DOI:** 10.3389/fendo.2021.672178

**Published:** 2021-05-31

**Authors:** Beata Rak, Maria Maksymowicz, Monika Pękul, Grzegorz Zieliński

**Affiliations:** ^1^ Department of Internal Diseases and Endocrinology, Public Central Teaching Hospital, Medical University of Warsaw, and the Laboratory of Experimental Medicine, Medical University of Warsaw, Warsaw, Poland; ^2^ Department of Pathology and Laboratory Diagnostics, Maria Sklodowska-Curie National Research Institute of Oncology, Warsaw, Poland; ^3^ Department of Neurosurgery, Military Institute of Medicine, Warsaw, Poland

**Keywords:** PitNET, DG, SG, densely and sparsely granulated pituitary tumors, corticotroph tumors, electron microscopy

## Abstract

**Purpose:**

Cushing’s disease is the most common cause of endogenous hypercortisolemia due to a corticotroph pituitary tumor. Up-to-date there is no reliable biomarker of invasiveness among corticotroph tumors, while it is well established in the literature that sparsely granulated somatotroph tumors are characterized by poorer prognosis. The aim of the study was to correlate multiple data including clinical, biochemical, radiological, and pathological findings (including granulation pattern) as well as immediate post-operative remission status among patients operated on due to corticotroph tumors.

**Methods:**

We enrolled all patients consecutively operated on for planned transsphenoidal neurosurgery due to corticotroph PitNETs in years 2010–2018. We excluded from analysis silent corticotroph tumors, plurihormonal PitNETs, and the Crooke’s cell adenomas.

**Results:**

We recorded 348 hormonally active corticotroph PitNETs. The results of the analysis showed the female predominance 79.88% (n = 278), with the mean age of Cushing’s disease occurrence 43.27 years of age. The mean time from the first signs and symptoms to the operation was 2 years. The women were diagnosed earlier (20–40 years of age *vs.* 50–60 years of age among men). We performed a detailed analysis of 277 cases classified by granularity pattern as DG or SG corticotroph PitNETs. Densely granulated tumors (DG) occurred four times more frequently than sparsely granulated (SG) (n = 225 *vs*. n = 52), at similar age (mean 42.94; median 40 *vs.* mean 45.46; median 45.5; p = 0.3896), but were characterized by lower Knosp’s scale grades (p = 0.0147*), smaller preoperative tumors’ volumes measured at MRI, and more commonly exhibited lower Ki-67 labeling index (<3%) (p = 0.0168*). What is more, DG adenomas more frequently achieved an immediate remission status (measured as postoperative cortisol concentration <2 µg/dl; p = 0.0180*), and the mean postoperative cortisol concentration in DG group was lower than in SG group (mean 5.375 µg/dl *vs*. 10.47 µg/dl; median 2.49 µg/dl *vs*. 6.52 µg/dl; p = 0.0028**).

**Conclusions:**

Our study indicates that DG corticotroph adenomas occurred at younger age, more commonly were microadenomas as compared to SG tumors, less frequently had invasive features in comparison to SG corticotroph adenomas (p = 0.0019**), and more commonly achieved an immediate postsurgical hormonal remission (p = 0.0180*). We highlight the need for an accurate differentiation of DG and SG subtypes in the pathomorphological diagnosis of corticotropic tumors, especially in invasive PitNETs.

## Introduction

Cushing’s disease firstly described in 1932 by Harvey Cushing is a life-threating condition caused by corticotroph pituitary tumor, and is known to be the most common cause of endogenous hypercortisolemia (1). The incidence has been estimated at 1.2 to 2.4 cases per million per year (2–5). It affects mostly female (estimated ratio female to male 3–4:1), nevertheless pediatric population exhibits trend towards men predominance (6, 7). The predisposition is approximately equal in age groups achieving puberty and reaches female inclination with age (6–8).

Cushing’s disease is associated with an increased mortality rate due to metabolic, cardiovascular, and infectious complications. It has been established that 50% of untreated patients die within 5 years of diagnosis (9). Neurosurgery remains the first line choice of the treatment with a long term follow up due to the high frequency of relapses (nearly 30% in long life follow-up) (9–11). Lindholm et al. demonstrated that persistent hypercortisolemia after initial neurosurgery among Cushing’s disease patients, was associated with five times higher standard mortality rate (SMR 5.06, p = <0.05) (2). Furthermore, several studies showed that negative effects on cardiovascular system sustained for several years even after biochemical remission have been achieved (12–14).

Despite the promotion by the 2004 WHO classification, the usefulness of the Ki-67 (MIB-1) labeling index and p53 nuclear immunoreactivity as reliable markers of aggressive pituitary tumors biology still remains controversial and imperfect (15, 16). Moreover, the most recent classification of tumors of the endocrine organs by the 2017 WHO classification (4th edition), excludes the term “atypical adenomas”, because of low predictive value and reproducibility. New definitions of “high risk” or “aggressive adenomas” are known to be: rapid growth of the tumor, radiological features of invasiveness, and high level of the Ki-67 proliferation index. Moreover, it has been established that apart from adenomas with elevated proliferative activity, some tumors’ subtypes predispose to aggressive behavior, i.e., sparsely granulated somatotroph tumors, silent corticotroph tumors, Crooke’s cell tumors, lactotroph tumors in men and plurihormonal Pit-1 positive tumors (17–19).

The 2017 World Health Organization classification of the tumors of the pituitary gland, among corticotroph tumors positive for ACTH and/or T-pit, distinguishes densely granulated corticotroph tumors (DG; most common), sparsely granulated corticotroph tumors (SG; less common) and Crooke’s cell tumors (the least common characterized by the poor prognosis) (18).

The aim of our retrospective study was to analyze available clinical data and pathological findings in the search of potential biomarkers of invasiveness among patients diagnosed with corticotroph pituitary tumors (pituitary neuroendocrine tumors, PitNETs) causing Cushing’s disease. We compared a global cohort of 348 patients with Cushing’s disease with a special interest and more detailed analysis for DG and SG phenotypes (n = 277). The results were predominantly obtained before the introduction of the 2017 WHO classification, therefore IHC staining for transcription factors was not performed.

## Material and Methods

### Patients’ Population

We enrolled into our retrospective analysis all patients consecutively operated on due to Cushing’s disease (n = 348) in years 2010–2018 at the Department of Neurosurgery, Military Institute of Medicine (Warsaw, Poland). The study was conducted in accordance with the Helsinki Declaration. All patients underwent full pre- and postoperative clinical, hormonal and imaging evaluation (including MRI).

The diagnosis of ACTH-dependent Cushing’s syndrome was established based on the clinical signs and symptoms, and standard hormonal criteria: increased excretion of urinary free cortisol (UFC), the lack of cortisol circadian rhythm (serum cortisol level above 7.5 µg/dl in the late-night hours), increased or detectable level of plasma ACTH at 08:00 and the failure of serum cortisol to suppress to less or equal than 1.8 µg/dl during the low dose dexamethasone suppression test (LDDST; 0.5 mg q.i.d. for 48 h). The Cushing’s disease was confirmed based on a serum cortisol and UFC suppression greater than 50% on high dose dexamethasone suppression test (HDDST; 2 mg q.i.d. for 48 h) and a positive result of stimulation test (increase in plasma cortisol level >20% above basal and serum ACTH level >50% above basal) with intravenous CRH injection (100 µg) and positive MR imaging for pituitary tumor as previously described in the literature (20, 21). In cases of negative or equivocal MRI results (intrasellar lesion ≤6 mm), bilateral inferior petrosal sinus sampling (BIPSS) was performed as a routine investigation tool. The study included only hormonally active corticotroph PitNETs causing symptomatic Cushing’s disease with the exclusion of silent corticotroph tumors, Crooke cell tumors and plurihormonal tumors.

### Postoperative Hormonal Assessment and Criteria for Remission

An immediate, postoperative remission was defined as a nadir morning serum cortisol level taken at 06:00 h on the first, second or third postoperative day lower or equal to 2 µg/dl. Early biochemical remission (six months after surgery) was recognized as a clinical and biochemical evidence or adrenal insufficiency or, in case of preserved adrenal function, biochemical evidence of eucortisolemia: UFC, morning serum cortisol and plasma ACTH levels within reference ranges, and preserved circadian rhythm of serum cortisol and ODST-induced serum cortisol suppression to ≤2 µg/dl. Serum cortisol levels were evaluated using electrochemiluminescence immunoassay (Elecsys 2010, Roche Diagnostics; sensitivity 0.02 μg/dl; reference range 6.2–19.4 μg/dl).

Preoperative MR imaging protocol included a T2-weighted 1,5T scanning in coronal sections and T1-weighted contrast enhanced and non-enhanced 1,5T scanning in coronal, sagittal and axial sections. Based on preoperative MR images, the cavernous sinus invasion was graded according to the Knosp’s scale (22) and was available in 311 cases (global cohort; including 243 DG and SG PitNETs for which EM was available). Subsequent, detailed analyses of invasiveness, tumor volume, Ki-67, and postoperative cortisol values were performed among DG and SG phenotypes only. To evaluate the volume of the tumors we used the formula height × length × width × π/6 measured in millimeters. All patients underwent microscopic transsphenoidal resection of pituitary adenomas performed by one neurosurgeon according to the identical surgical protocol. After neurosurgery, patients were followed-up till the 1st of January 2019 (clinical, biochemical, and MRI evaluation). The median follow-up of the study was 54 months (mean 50.7 months; SD ± 30.98 months). An appropriate medical intervention was provided if needed. During the follow-up, 24 cases (6.9%) of tumor recurrence were recorded among all hormonally active corticotroph PitNETs (we have found eight SG, eight DG and five Crook’s cell adenomas, for the others the tissue for ME was not possible to obtain).

### Histology and Immunohistochemistry (IHC)

The pathological diagnosis of pituitary adenomas (PitNETs) was performed by one pathologist at the Department of Pathology and Laboratory Diagnostics, Maria Sklodowska-Curie National Research Institute of Oncology, Warsaw, Poland. Pathological evaluation for pituitary tumors (PitNETs) included routine immunohistochemical staining on GH, PRL, ACTH, β-TSH, β-FSH, β-LH, alpha subunit, and Ki-67. In selected cases, immunostaining was extended to the expression of Cam5.2, SSTR2A, SSTR5, p53, MGMT, Collagen IV and others, when appropriate.

The standard immunohistochemical evaluation was performed using 10% buffered formalin, in which tissue samples were embedded and stained with hematoxylin and eosin. Immunohistochemical staining was performed on paraffin-embedded sections according to the labeled EnVision Flex Visualization System (K8000, Dako/Agilent) with DAB (3,3′-diaminobenzidine). For the ACTH evaluation we used antibody from Thermo Fisher Scientific (concentration 1:500; cat. no.: M5-1452-P1) and Ki-67 from Dako company (MIB-1 clone; ready to use antibody) as well as Anti-Cytokeratin antibody from Ventana company (Cam 5.2, cat. no.: 790-4555).

### Electron Microscopy (EM)

For 277 patients with Cushing’s disease (79.6%), electron microscopy was also performed. In other cases, material for EM was not possible to obtain. Small pieces of pituitary tumor (PitNETs) tissues were fixed using 2.5% glutaraldehyde, postfixed in 1% osmium tetroxide, dehydrated with ethanol and propylene oxide and subsequently embedded in epoxy resin (Epon 812). Ultrathin sections were counterstained with uranyl acetate and lead citrate followed by examination with a Philips CM120 BioTWIN transmission electron microscope. Corticotroph tumors were classified on the basis of commonly accepted histological and ultrastructural features, such as densely granulated (DG) or sparsely granulated (SG) (23), and only those were included into subsequent analysis. We excluded Crooke cell tumors (n = 16).

### Statistical Analysis

Methods of descriptive statistics (mean, median, standard deviations) were employed. Statistical tests: Chi², Fisher’s exact test, Spearman’s correlation, Kruskal–Wallis, and nonparametric Mann–Whitney, were used when appropriate. A p-value of <0.05 was considered as statistically significant. All calculations were performed using Excel (Microsoft Office), Statistica (StatSoft) and GraphPad Prism 8 (GraphPad Software Inc.).

## Results

### General Cohort of 348 Patients With Cushing’s Disease

**Figure 1:**
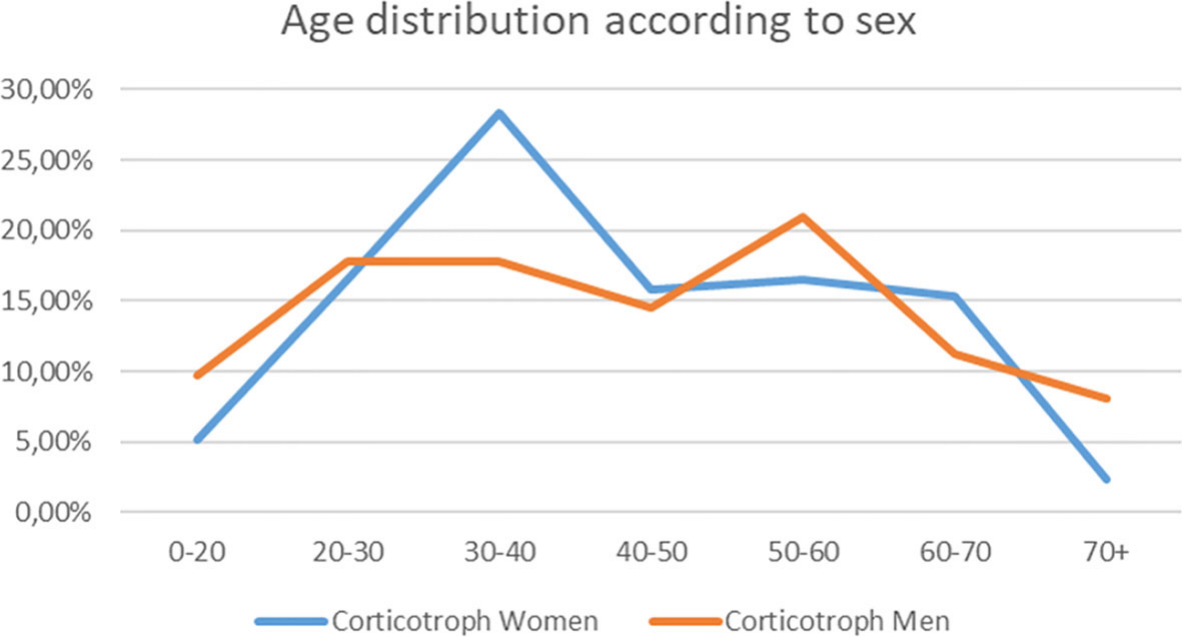
The graph shows the frequency of Cushing’s disease diagnosis in particular decades of life for women (blue line) and men (orange line). The percentages contribute to total number of diagnoses for men and women separately.

In our retrospective analysis women dominated accounting for 278 cases out of 348 (79.88%) (F:M ratio was 3.97:1). The mean age at the time of diagnosis for Cushing’s disease was 43.45 years (SD ± 16.23). The corticotroph tumors among women were diagnosed earlier (mean age among women 43.27 years, SD ± 15.66, median 40; *vs.* mean age 44.24, SD ± 18.45, median 43.5 among men), and occurred predominantly between the 2nd and 4th decades of life compared to the 5th and 6th decades in men. A detailed graphical presentation of the age distribution is presented in **Figure 1**.

The mean time from the first signs and symptoms to the operation was 2 years (range from 1 month to 20 years). We correlated the Knosp’s scale grades with the patients’ age at the time of the diagnosis, as well as with the time from the onset of symptoms in years. The results are shown in **Supplementary Figure 1**. Cushing’s disease was predominantly diagnosed at early Knosp’s grades as compared to other PitNETs in our previous study (24).

### Comparison of DG and SG Corticotroph Phenotypes in 277 Patients

**Figure 2:**
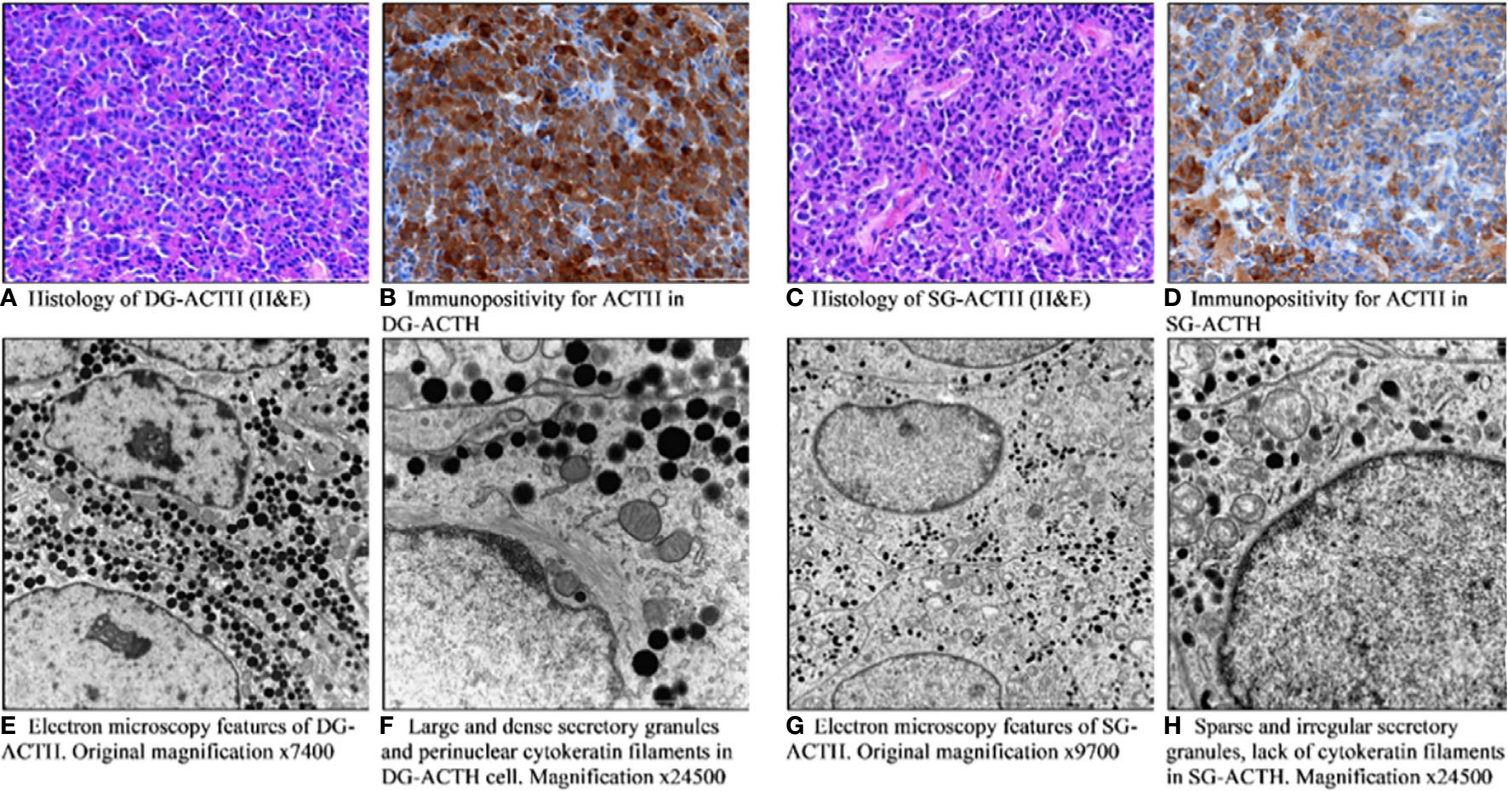
Pathomorphological features of densely **(A, B, E, F)** and sparsely granulated **(C, D, G, H)** corticotroph pituitary tumors. **(A)** Histology of DG-ACTH (H&E). **(B)** Immunopositivity for ACTH in DG-ACTH. **(C)** Histology of SG-ACTH (H&E). **(D)** Immunopositivity for ACTH in SG-ACTH. **(E)** Electron microscopy features of DG-ACTH. **(F)** Large and dense secretory granules and perinuclear cytokeratin filaments in DG-ACTH cell. **(G)** Electron microscopy features of SG-ACTH. **(H)** Sparse and irregular secretory granules, lack of cytokeratin filaments in SG-ACTH.

Sparsely and densely granulated pituitary tumors were established based on characteristic histological and ultrastructural features in EM (n = 277). The sparsely granulated tumors are characterized by chromophobic cells. PAS staining is faintly positive, likewise immunostaining for ACTH which is usually focal and weak. In contrast, densely granulated corticotroph tumors are composed of basophilic cells, which have a strong and diffuse expression of ACTH as well as strong positive PAS staining. In the vast majority of DG tumors, cells resemble normal corticotrophs: secretory granules are usually numerous, electron-dense, spherical, or irregular in shape, reaching approximately 300–700 nm in size. An important feature is the presence of perinuclear bundles of cytokeratin fibers; the Golgi complex and the rough endoplasmic reticulum are moderately developed. In contrast, the SG tumor cells consist of poorly developed cytoplasmic organelles, small and sparse secretory granules with a diameter of 150 to 250 nm, and do not contain cytokeratin fibers around the nuclei. The representative pictures of DG and SG corticotroph tumors are shown in **Figure 2**.

Likewise in general cohort of patients with Cushing’s disease, in both phenotypes women dominated (see **Table 1**). We observed that DG PitNETs occurred at similar age as SG tumors (mean 42.94; median 40 years of age *vs.* mean 45.46; median 45.5 years of age; p = 0.3896), and accounted for majority of microadenomas cases, what is shown in **Table 1**.

**Table 1:** Clinical and tumoral differences between DG and SG corticotroph tumors in 277 patients with Cushing’s disease.

Morphotype	DG	SG	p -value
**Number Total**	**225**	**52**	** **
(%)	81.23%	18.77%	** **
**Sex**			
Women	**189 (84%)**	**38 (73.08%)**	p = 0.0649^1^
Men	**36 (16%)**	**14 (26.92%)**	
**Mean age (** ± SD)	**42.94 (**16.06)	**45.46 (**16.87)	NS
Median age	40	45.5	p = 0.3896^3^
**Tumor size (n = 243)**			
Microadenomas	**113**	**17**	p = 0.0290*^1^
	56.78% (113/199)	38.63% (17/44)	
Macroadenomas	**86**	**27**	
	43.21% (86/199)	61.36% (27/44)	
**Preoperative MRI**			
**(n = 243)**	**33**	**17**	p = 0.0019**^2^
Invasion (Knosp’s ≥2)	16.6% (33/199)	38% (17/44)	
**Ki-67 index (n = 221)**			
≥3%	22.2% (40/180)	41.5% (17/41)	p = 0.0168*^2^
< 3%	77.8% (140/180)	58.5% (24/41)	
**Immediate remission (n = 235)**	**92**	**12**	p = 0.0180*^2^
(cortisol <2µg/dl)	47.9% (92/192)	27.9% (12/43)	

NS, not significant; *p < 0.05; **p < 0.01. Bolding was used in order to highlight the major findings.

**Figure 3:**
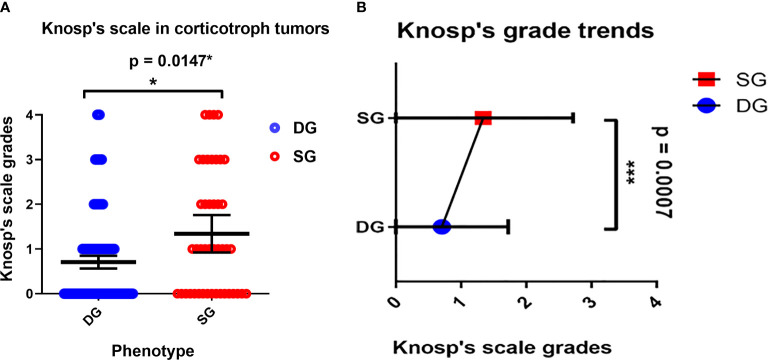
**(A)** The Distribution of DG and SG subtypes in corticotroph tumors according to the Knosp’s scale grades. Blue circles represent single cases of DG corticotroph tumors, and red circles SG corticotroph tumors. Calculated according to Chi² test (p = 0.0147*). The error bars show mean with 95% CI. **(B)** The overall trend among all corticotroph PitNETs exhibits higher Knosp’s scale grades among SG corticotroph tumors. Blue color represents mean with 95% CI for DG corticotroph tumors and red color for SG corticotroph tumors. Calculated according to Chi² test for trends p = 0.0007***.

**Figure 4:**
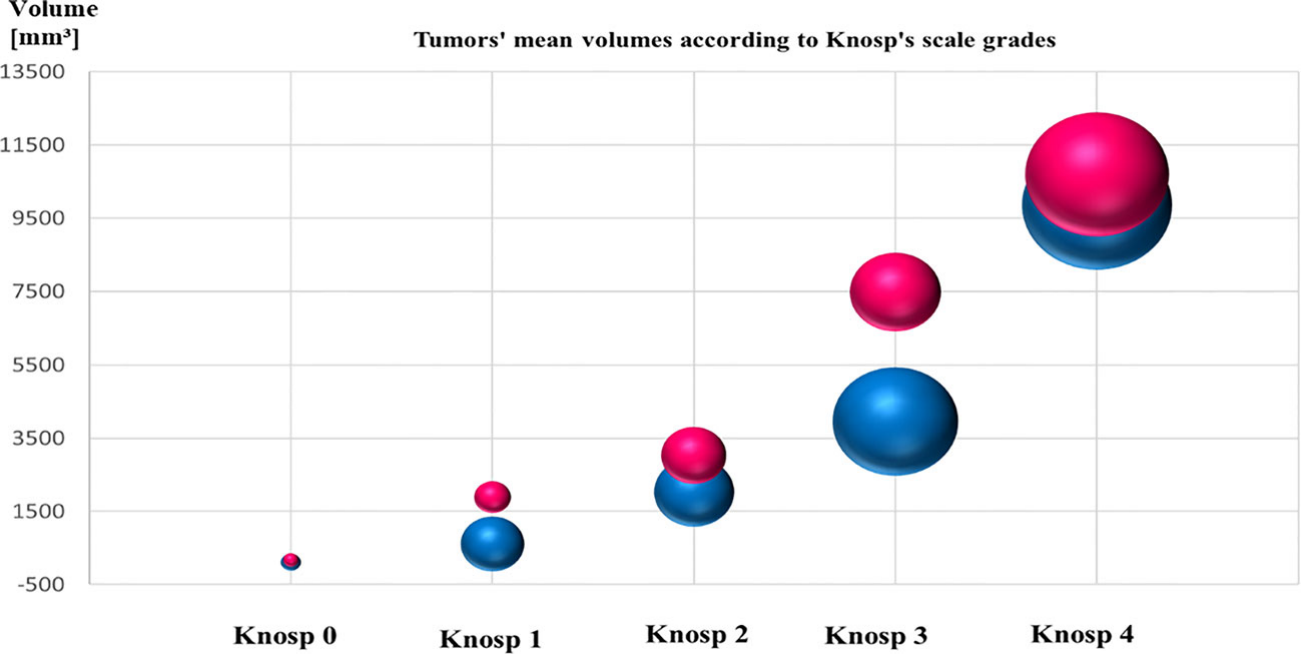
The graph shows mean tumors’ volume of SG and DG corticotroph PitNETs, and Knosp’s scale grades. Red color represents SG tumors and blue color DG tumors. The size of the spheres corresponds to the numbers of diagnosed cases. SG phenotypes reached higher mean volume of the tumors compared to DG corticotroph tumors. Tumors’ volumes were calculated according to formula height × length × width × π/6 measured in millimeters on preoperative MRI scans.

The Knosp’s scale grades were established based on preoperative MRI, as previously described in the literature (22) and were available for 243 cases among patients with established granularity pattern. We observed increased frequency of SG tumors among higher Knosp’s scale grades (2–4). The exact participation of DG and SG tumors for particular Knosp’s scale grades are presented in **Figure 3A**. Furthermore, the tumor volumes’ were higher among SG tumors compared to DG ones (see **Figure 4**). Likewise, invasiveness defined as a Knosp’s scale grade at least two occurred more frequently in SG corticotroph tumors (p = 0.0019**). Moreover, SG corticotroph tumors presented a significantly higher proliferation index (p = 0.0168*) in comparison to DG corticotroph tumors (see **Table 1**).

**Figure 5:**
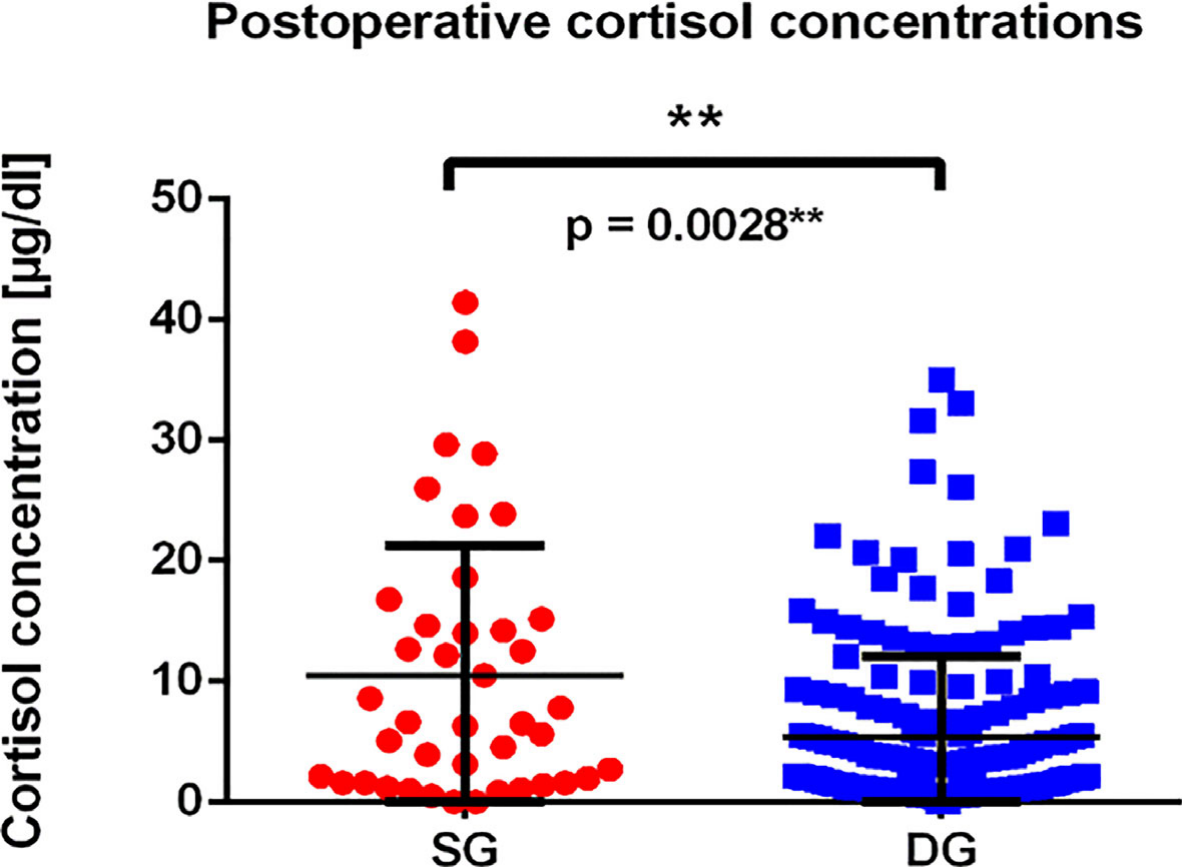
The graph presents postoperative cortisol concentrations among patients with SG and DG corticotroph tumors. Each point represents single value of measurement for SG (red dots), and DG (blue squares) tumors; p = 0.0028** calculated using Mann–Whitney test.

Subsequently, we analyzed the morning serum cortisol concentration during the first days after surgery (**Figure 5**). We have found, that mean cortisol concentration in SG group was higher than in the DG group, reaching 10.47 µg/dl in SG group (median 6.52 µg/dl) compared to 5.375 µg/dl in DG group (median 2.49 µg/dl). The difference was statistically significant (p = 0.0028**). What is more, DG tumors more frequently achieved an immediate remission status defined as cortisol level below 2 µg/dl (p = 0.0180*), which is shown in **Table 1**.

## Discussion

It is worth highlighting that the EM in the evaluation of granularity pattern is not routinely used nowadays due to its time-consuming procedure, expensive costs as compared to IHC which is cheaper and widely available. Thus in most pathology departments, ME has become replaced by IHC, which also enables the differentiation between DG and SG tumors. Nevertheless, in cases of weak or only focal ACTH staining (which may also be due to technical reasons), EM can be very helpful. Fortunately, we still have the possibility to perform EM routinely. This is a reason why in our series, EM was performed in all cases in which the material was fixed and preserved for the EM technique (277 out of 348). In our material, there were about 8–10% of cases that raised doubts based on IHC, and the verification of diagnosis in EM was extremely useful.

The term sparsely or densely granulated refers to the cytoplasmic appearance of hormone-containing secretory granules in immunohistochemistry and/or density of secretory granules in electron microscopy (25). A study by Founger et al. reported, that sparsely granulated somatotroph tumors were characterized by a worse response to somatostatin analogs and had a lowest immunohistochemical expression of E-cadherin, a marker of dedifferentiation (26). Larkin et al. showed, that the sparsely granulated somatotroph tumors compared to densely granulated ones were larger, more invasive, occurred more commonly in younger age, and had decreased response to octreotide suppression test. Further, the authors suggested, that histological phenotype of SG somatotroph tumors but not genotype refers more accurately to both clinical and biochemical characteristics as well as response to the octreotide suppression test (27). Independently, other studies confirmed that sparsely granulated phenotype is associated with worse response to somatostatin analogues therapy as well as poorer outcome (28–30).

In terms of corticotroph PitNETs, both DG and SG corticotroph tumors reveal strong expression of T-pit and low molecular weight keratin (LMWK) (25, 31–34). In the review by Mete and Asa, the authors highlighted clinical differences between DG and SG corticotroph tumors (25). They proposed the terms: “small tumor, big Cushing” for densely granulated lesions and “big tumor, small Cushing” for sparsely granulated ones. The finding was also observed by Witek et al. (35).

Although limited cases have been published on granularity patterns and invasiveness among corticotroph tumors, our current study on the large patients’ series with Cushing’s disease also suggests more invasive behavior of sparsely granulated corticotroph tumors (35, 36). Witek et al. reported that greater tumor volumes were associated with higher Ki-67 among SG corticotroph PitNETs on a group of 59 patients (35). Another study performed among 41 corticotroph tumors, showed that both phenotypes occurred equally among men and women, and at similar age groups with alike biochemical presentation, but SG granulated corticotroph tumors reached larger sizes (36). But yet, the retrospective analysis of 15 years’ experience revealed that two out of four reported cases of pituitary carcinomas were initially diagnosed as sparsely granulated corticotroph tumors (36). There exist reports of transformation from DG to SG corticotroph tumors with subsequent metastases to the liver rising a diagnosis of pituitary carcinoma (37). In our series, we observed one patient with pituitary carcinoma originating from Crooke’s cell tumor, but we did not observe any case of pituitary carcinoma derived from neither SG nor DG corticotroph tumors.

In this large cohort, DG corticotroph PitNETs were more frequent than SG, as in two other studies (36, 38). According to Dogansen et al. (36), we found no statistically significant difference in sex and age between both phenotypes. In contrast, Mete et al. (38) stressed that DG occurred at younger age (44 *vs.* 52 years). In our cohort, the difference (42.94 *vs.* 45.46) was not statistically significant (p = 0.3896). We confirmed that DG tumors were in majority microadenomas and that SG tumors were larger and more frequently invasive (38%; p = 0.0019**) reaching both higher Knosp’s scale grades, and tumor volumes (see **Figures 3**, **4** and **Table 1**).

Moreover, among SG phenotype, Ki-67 proliferation index more often achieved 3% compared to DG. Finally, DG corticotroph tumors more frequently than SG corticotroph tumors reached an immediate remission status defined as cortisol level lower than 2 µg/dl (p = 0.0180*), but yet cortisol concentrations measured postoperatively were significantly higher in SG phenotypes (p = 0.0028**). In contrast, Dogansen et al. observed similar remission rate between DG and SG, however, the research was performed on a smaller group of 41 corticotroph PitNETs (36). The exact mechanisms underlying the pathogenesis of invasive corticotroph tumors remain unclear and are still being investigated. The most recent transcriptomic data focuses on the role of microRNAs and their potential mRNA targets (39–42). For the time being, however, they are not considered as biomarkers of invasiveness either tumor remission in the WHO classification in any type of PitNETs.

To the best of our knowledge, this analysis contains one of the biggest cohort of DG and SG corticotroph tumors, performed in a single-center. We highlight the importance of granularity pattern among corticotroph pituitary tumors, since the SG phenotypes correlate with more invasive features such as higher Knosp’s scale grades, bigger tumor volumes, and higher Ki-67 proliferation index. But yet, SG phenotypes are less frequently prone to achieve an immediate remission status. The obtained results justify the need for an accurate differentiation of DG and SG subtypes in the pathomorphological diagnosis of corticotropic tumors, especially in invasive PitNETs. The limitation of our analysis was the lack of the exact cortisol values in a long-term follow-up after surgery as well as the lack of some parameters (mitotic count and p53 staining) which could allow us to classify SG and DG tumors according to the most recently proposed by Trouillas et al. French five-tiered prognostic classification (43). Nevertheless, to ultimately define the role of granularity pattern in a correlation with remission status, it is crucial to evaluate cortisol concentration in a prospective study with a long-term follow-up. This issue needs further investigation and will be addressed in the future study. Besides, it would be desirable to evaluate disease relapse in relation to the granulation pattern.

## Data Availability Statement

The original contributions presented in the study are included in the article/**Supplementary Material**. Further inquiries can be directed to the corresponding author.

## Ethics Statement

The studies involving human participants were reviewed and approved by The Bioethical Committee at the Medical University of Warsaw. Written informed consent for participation was not required for this study in accordance with the national legislation and the institutional requirements.

## Author Contributions

BR**—**study design, literature search, retrospective data collection in years 2010–2018, data analysis and figure preparation, and writing the manuscript. MM—study design, performing all pathomorphological evaluations, data collection during the whole study, figure preparation, and revising the manuscript critically for important intellectual content. MP**—**assistance in pathomorphological evaluations and data collection during the study. GZ—study design, performed all neurosurgical operations, data collection during the whole study, revising the manuscript critically for important intellectual content, supervising the work, and supporting at each step of the study. All authors contributed to the article and approved the submitted version.

## Funding

This manuscript was prepared during the financing obtained by the author BR as doctoral scholarship Etiuda funded by the National Science Center of Poland UMO-2018/28/T/NZ5/00304, and grant Preludium funded by the National Science Center of Poland UMO-2016/23/N/NZ5/02597.

## Conflict of Interest

The authors declare that the research was conducted in the absence of any commercial or financial relationships that could be construed as a potential conflict of interest.
