# Supplementary material for: Clinical, Biological, Radiological Pathological and Immediate Post-Operative Remission of Sparsely and Densely Granulated Corticotroph Pituitary Tumors: A Retrospective Study of a Cohort of 277 Patients With Cushing’s Disease
Source: Front Endocrinol (Lausanne). 2021 May 31;12:672178. doi: 10.3389/fendo.2021.672178 (PMC8202403; doi:10.3389/fendo.2021.672178)

**Supplementary Figure 1.** Fig A shows the correlations between Knosp’s grades and the age of patients at the time of surgery (r=0.1702, p=0.005**). Fig B presents the correlations between Knosp’s grades and the time from the onset of symptoms to the final diagnosis (r=-0.09954, p=0.102). *Calculated according to Spearman’s correlation coefficiency.*

**A B**


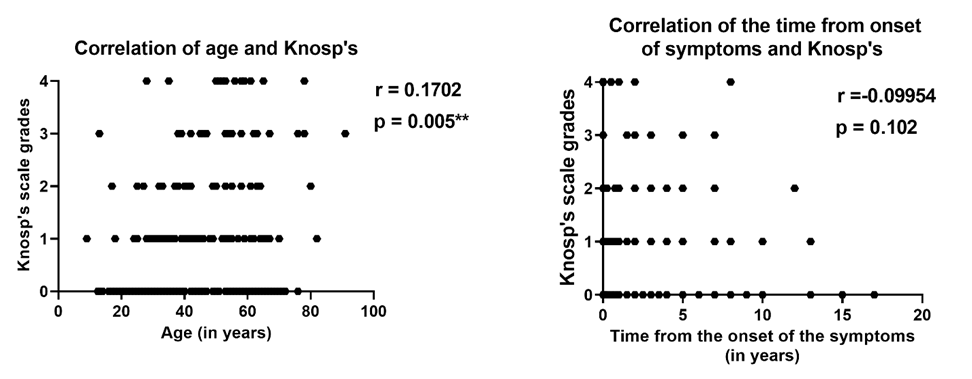

Supplement: Supplementary file 1 [file DataSheet_1.docx]
